# Supplementary material for: A model for the intrinsic limit of cancer therapy: Duality of treatment-induced cell death and treatment-induced stemness
Source: PLoS Comput Biol. 2022 Jul 25;18(7):e1010319. doi: 10.1371/journal.pcbi.1010319 (PMC9352192; doi:10.1371/journal.pcbi.1010319)
Supplement: S1 Text — (ZIP) [file pcbi.1010319.s001.zip › S1_Text.pdf]

S1 Text for "A model for the intrinsic limit of cancer therapy: duality of treatment-induced cell death and treatment-induced stemness"

Erin Angelini[1], Yue Wang[1,2], Joseph Xu Zhou[3,4], Hong Qian[1], Sui Huang[4] *

1 Department of Applied Mathematics, University of Washington, Seattle, WA 98195
2 Institut des Hautes Études Scientifiques, 91440 Bures-sur-Yvette, France
3 Immuno-Oncology Department, Novartis Institutes for BioMedical Research, Cambridge, MA 02139
4 Institute for Systems Biology, Seattle, WA 98109

* Corresponding author.
Name: Sui Huang
E-mail: sui.huang@isbscience.org
ORCiD: 0000-0002-3545-4665

Linear stability analysis

By the general theory of linear ODEs, the exact solution to this system is

$$x(t) = c_1 e^{\lambda_1 t} v^{(1)} + c_2 e^{\lambda_2 t} v^{(2)}\,, \tag{S1}$$

where $\lambda_i, v^{(i)}$ are the right eigenpairs of A. The eigenvalues of A are given by:

$$\lambda_{1,2} = \frac{g_S + g_R - k_{SR} - k_{RS} \pm \sqrt{(g_S + g_R - k_{SR} - k_{RS})^2 - 4(g_S g_R - g_R k_{SR} - g_S k_{RS})}}{2} \tag{S2}$$

where we define the net growth rates $g_S = b_S - d_S$and $g_R = b_R - d_R$ for simplicity.

Define $\alpha = g_R - g_S - (k_{RS} - k_{SR})$ and $\beta = \sqrt{\alpha^2 + 4k_{SR}k_{RS}}$. Using the expression given in S2 Eq, along with the fact that

$$g_S + g_R - k_{SR} - k_{RS} = \alpha + 2(g_S - k_{SR}),$$

we can write λ_1 and λ_2 in terms of α and β:

$$\begin{aligned}
\lambda_{1,2} &= \frac{\alpha + 2(g_S - k_{SR}) \pm \sqrt{(\alpha + 2(g_S - k_{SR})^2 - 4(g_S g_R - g_R k_{SR} - g_S k_{RS})}}{2} \\
&= \frac{\alpha + 2(g_S - k_{SR}) \pm \sqrt{\alpha^2 + 4(\alpha - g_R)(g_S - k_{SR}) + 4(g_S - k_{SR})^2 + 4g_S k_{RS}}}{2} \\
&= \frac{\alpha + 2(g_S - k_{SR}) \pm \sqrt{\alpha^2 + 4(g_S - k_{SR})(\alpha - g_R + g_S - k_{SR}) + 4g_S k_{RS}}}{2} \\
&= \frac{\alpha + 2(g_S - k_{SR}) \pm \sqrt{\alpha^2 - 4k_{RS}(g_S - k_{SR}) + 4g_S k_{RS}}}{2} \\
&= \frac{\alpha + 2(g_S - k_{SR}) \pm \sqrt{\alpha^2 + 4k_{SR}k_{RS}}}{2} \\
&= \frac{\alpha + 2(g_S - k_{SR}) \pm \beta}{2}\,.
\end{aligned}$$

Because we assume that k_{SR} and k_{RS} are strictly positive, the discriminant $\alpha^2 + 4k_{SR}k_{RS}$ is strictly positive, and $\beta > 0$. Therefore, both of the above eigenvalues are real and distinct. The corresponding eigenvectors are

$$v^{(1,2)} = \frac{1}{2k_{SR}} \begin{bmatrix} -\alpha \pm \beta \\ 2k_{SR} \end{bmatrix} .$$

The coefficients c_1, c_2 in S1 Eq satisfy the linear system $x(0) = Vc$, where V is the matrix whose columns are the eigenvectors of A, and $c = \begin{bmatrix} c_1 & c_2 \end{bmatrix}^T$. It can be shown that

$$c_1 = \frac{k_{SR}}{\beta} x_1(0) + \frac{\alpha + \beta}{2\beta} x_2(0), \quad \text{and} \quad c_2 = -\frac{k_{SR}}{\beta} x_1(0) - \frac{\alpha - \beta}{2\beta} x_2(0) .$$

In the following analysis, we require that $g_R > 0$ under drug treatment as a general definition of the drug-resistant phenotype.

The origin is guaranteed to be a saddle point wherever

$$g := \frac{g_S}{g_R} > \frac{g_S - k_{SR}}{k_{RS}} \tag{S3}$$

The condition in S3 Eq has two interpretations based on whether $g_S > 0$ or $g_S < 0$. Wherever $g_S > 0$, S3 Eq requires that at least one of the relative fitness of the drug-sensitive phenotype g and the backflow rate k_{RS} are sufficiently large compared to the net flux $g_S - k_{SR}$. Wherever $g_S < 0$, S3 Eq is true when the ratio of the net flux to the backflow rate is larger in magnitude than the relative fitness.

If instead the growth and transition rates satisfy the inequality

$$g = \frac{g_S}{g_R} < \frac{g_S - k_{SR}}{k_{RS}} \tag{S4}$$

then the relationship between $g_S + g_R$ and $k_{SR} + k_{RS}$ determines whether the origin is stable or unstable (Table A). When $g_S + g_R > k_{SR} + k_{RS}$, the origin is an unstable node, and the tumor grows exponentially (Table A). On the other hand, when $g_S + g_R < k_{SR} + k_{RS}$, the origin is a stable node, and the tumor tends toward extinction, i.e., treatment has eradicated the tumor (Table A).

One way to frame these two cases is to consider which of the two dynamics, growth or phenotype switching, is the dominant force behind the overall population dynamics. Considering the growth of the overall population of cells, we can think of $g_S + g_R$ as the unweighted growth rate of the tumor, as the true growth rate is given by $\frac{dx_1}{dt} + \frac{dx_2}{dt} = g_S x_1 + g_R x_2$. Considering only the pure phenotype switching dynamics, where total size is fixed, the quantity $k_{SR} + k_{RS}$ is the relaxation time of these dynamics. Therefore, when pure growth outweighs pure switching, tumor growth is unchecked, and no tumor control is possible. If, however, the switching dynamics dominate the underlying growth rate, the tumor will eventually be eradicated.

Table A. Summary of all possible cases for the stability of the origin under the ODE in Eq 1.

	$g_S + g_R > k_{SR} + k_{RS}$	$g_S + g_R < k_{SR} + k_{RS}$
$g > \frac{g_S - k_{SR}}{k_{RS}}$	**saddle point**	**saddle point**
$g < \frac{g_S - k_{SR}}{k_{RS}}$	**unstable node**	**stable node**

Dynamics of tumor population recovery

The following analysis follows from the fact that we can express the total population $N(t) = x_1(t) + x_2(t)$ as a linear combination of exponential terms via the exact solution $x(t)$:

$$N(t) = x_1(t) + x_2(t) = c_1\big(v_1^{(1)} + v_2^{(1)}\big)e^{\lambda_1 t} + c_2\big(v_1^{(2)} + v_2^{(2)}\big)e^{\lambda_2 t}\,. \tag{S5}$$

We can use the exact solution given in S5 Eq to derive an approximate expression for t_P, assuming that it is non-zero. If $N'(0) < 0$, $t = t_P$ is the unique non-zero time point for which $N(t) = N(0)$. Using S5 Eq, the equation $N(t_P) = N(0)$ reads:

$$N(t_P) \equiv c_1\big(v_1^{(1)} + v_2^{(1)}\big)e^{\lambda_1 t_P} + c_2\big(v_1^{(2)} + v_2^{(2)}\big)e^{\lambda_2 t_P} = N(0)\,. \tag{S6}$$

Solving the above expression analytically for t_P is not possible, as we have established that the eigenvalues $\lambda_{1,2}$ are distinct.

Under the assumption that the exponential term $e^{\lambda_1 t}$ gives the dominant contribution to $N(t)$ at time $t = t_P$, we get:

$$N(t_P) \approx c_1\big(v_1^{(1)} + v_2^{(1)}\big)e^{\lambda_1 t_P} =: N^*\,. \tag{S7}$$

Setting the above expression equal to $N(0)$ and solving for t_P, we obtain the following approximation t_P^* of t_P :

$$t_P \approx t_P^* := \frac{1}{\lambda_1}\ln\left[\frac{N(0)}{c_1\big(v_1^{(1)} + v_2^{(1)}\big)}\right]. \tag{S8}$$

Taking the absolute value of the difference between $N(t_P)$ and its approximation $N^* := N(t_P^*)$ as a proxy for the absolute error $|t_P - t_P^*|$, we find that the error of the approximation in S7 Eq scales exponentially with the negative eigenvalue λ_2:

$$|N(t_P) - N^*| = \Big|c_2\big(v_1^{(2)} + v_2^{(2)}\big)\Big|e^{\lambda_2 t_P}. \tag{S9}$$

In the above derivation, we neglected the negative eigenvalue term to obtain a closed-form approximation for t_P. However, the rate of cancer remission is equally as important in setting the TTP as is the rate of cancer regrowth. Thus, in order to better understand how t_P changes with m, we also consider the point $t = t_{min}$ at which the total population $N(t)$ reaches its global minimum. Unlike with t_P, we can determine a closed form of t_{min} in terms of the model parameters. The turning point of $N(t)$ occurs when

$$0 = \frac{dN}{dt} \equiv g_S x_1(t) + g_R x_2(t) \iff \rho(t) := \frac{x_2(t)}{x_1(t)} = -\frac{g_S}{g_R}\,.$$

Thus, in order to better understand the dynamics of $N(t)$, we must first understand those of the ratio $\rho(t)$ of drug-resistant cells to drug-sensitive cells.

Using the quotient rule, we can compute the derivative of this ratio, $\frac{d\rho}{dt}$:

$$\frac{d\rho}{dt} = \frac{x_1x_2' - x_2x_1'}{x_1^2} = \frac{x_2}{x_1}\left(\frac{x_2'}{x_2} - \frac{x_1'}{x_1}\right) = \rho\left(g_R - k_{RS} + \frac{k_{SR}}{\rho} - g_S + k_{SR} - k_{RS}\rho\right).$$

Recall that $\alpha = g_R - g_S - (k_{RS} - k_{SR})$. Then the above expression simplifies to

$$\frac{d\rho}{dt} = -k_{RS}\rho^2 + \alpha\rho + k_{SR}\,. \tag{S10}$$

This ODE is a Riccati equation with initial condition $\rho_0 := \rho(0) = x_2(0)/x_1(0)$. Define $q(\rho)$ as the polynomial on the right-hand side of S10 Eq:

$$q(\rho) = -k_{RS}\rho^2 + \alpha\rho + k_{SR}\,.$$

As long as $q(\rho)$ has real roots, we can solve S10 Eq analytically via separation of variables. Recall the definition $\beta = \sqrt{\alpha^2 + 4k_{SR}k_{RS}}$. Then the roots of $q(\rho)$ are given by

$$\rho_{1,2} = \frac{-\alpha \pm \beta}{-2k_{RS}} = \frac{1}{2k_{RS}} (\alpha \mp \beta) \ .$$

We know that $\alpha^2 + 4k_{SR}k_{RS} > 0$, which tells us that the roots $\rho_{1,2}$ are real and distinct. Therefore, we can write $q(\rho)$ in the following factored form:

$$q(\rho) = -k_{RS}(\rho - \rho_1)(\rho - \rho_2).$$

Using the fact that $\rho_1 < \rho_2$, a simple phase-line stability analysis tells us that ρ_1 is an unstable fixed point, and ρ_2 is a stable fixed point.

With this factorization of $q(\rho)$, we can now solve the given IVP to obtain a closed form of $\rho(t)$. We begin by deriving the partial fraction decomposition of $1/q(\rho)$:

$$\begin{aligned}
&\frac{1}{q(\rho)} = \frac{1}{-k_{RS}(\rho - \rho_1)(\rho - \rho_2)} = \frac{C_1}{\rho - \rho_1} + \frac{C_2}{\rho - \rho_2} \\
&\iff 1 = -k_{RS}C_1(\rho - \rho_2) - k_{RS}C_2(\rho - \rho_1) \iff -\frac{1}{k_{RS}} = (C_1 + C_2)\rho - C_1\rho_2 - C_2\rho_1 \\
&\iff C_1 + C_2 = 0 \quad \text{and} \quad \frac{1}{k_{RS}} = C_1\rho_2 + C_2\rho_1 \\
&\iff \frac{1}{k_{RS}(\rho_2 - \rho_1)} = C_1 = -C_2.
\end{aligned}$$

The unique solution to the above system of linear equations for C_1 and C_2 is

$$C_1 = -C_2 = \frac{1}{k_{RS}(\rho_2 - \rho_1)} \ .$$

Therefore, the partial fraction decomposition of $1/q(\rho)$ is given by

$$\frac{1}{q(\rho)} = \frac{1}{k_{RS}(\rho_2 - \rho_1)} \left(\frac{1}{\rho - \rho_1} - \frac{1}{\rho - \rho_2} \right) \ .$$

We can now solve the Riccati equation in S10 Eq for $\rho(t)$:

$$\frac{d\rho}{dt} = q(\rho) \iff \int \frac{d\rho}{q(\rho)} = \int dt \, ,$$

where we use the partial fraction decomposition of $1/q(\rho)$ to evaluate the integral in ρ up to a constant of integration:

$$\int \frac{d\rho}{q(\rho)} = \frac{1}{k_{RS}(\rho_2 - \rho_1)} \int \left(\frac{1}{\rho - \rho_1} - \frac{1}{\rho - \rho_2} \right) d\rho = \frac{1}{k_{RS}(\rho_2 - \rho_1)} \ln \left(\frac{\rho - \rho_1}{\rho - \rho_2} \right) - C.$$

It follows from this result that

$$\frac{1}{k_{RS}(\rho_2 - \rho_1)} \ln \left(\frac{\rho - \rho_1}{\rho - \rho_2} \right) = t + C \, . \tag{S11}$$

Applying the initial condition $\rho(0) = \rho_0$, we can solve for the constant C:

$$C = \frac{1}{k_{RS}(\rho_2 - \rho_1)} \ln \left(\frac{\rho_0 - \rho_1}{\rho_0 - \rho_2} \right) \ .$$

We can now solve for the exact solution ρ in S11 Eq:

$$
\begin{aligned}
&\ln\left(\frac{\rho-\rho_1}{\rho-\rho_2}\right) = k_{RS}(\rho_2-\rho_1)t + \ln\left(\frac{\rho_0-\rho_1}{\rho_0-\rho_2}\right)\\
\iff &\frac{\rho-\rho_1}{\rho-\rho_2} = \frac{\rho_0-\rho_1}{\rho_0-\rho_2}e^{k_{RS}(\rho_2-\rho_1)t}\\
\iff &\rho\Big(\rho_0-\rho_2-(\rho_0-\rho_1)e^{k_{RS}(\rho_2-\rho_1)t}\Big) = \rho_1(\rho_0-\rho_2)-\rho_2(\rho_0-\rho_1)e^{k_{RS}(\rho_2-\rho_1)t}\\
\iff &\rho(t) = \frac{\rho_1(\rho_0-\rho_2)-\rho_2(\rho_0-\rho_1)e^{k_{RS}(\rho_2-\rho_1)t}}{\rho_0-\rho_2-(\rho_0-\rho_1)e^{k_{RS}(\rho_2-\rho_1)t}}\,.
\end{aligned}
$$

From this closed expression, we find an explicit expression for t_{min} by solving the equation $\rho(t) = -g_S/g_R$:

$$
\begin{aligned}
&\rho(t) = \frac{\rho_1(\rho_0-\rho_2)-\rho_2(\rho_0-\rho_1)e^{k_{RS}(\rho_2-\rho_1)t}}{\rho_0-\rho_2-(\rho_0-\rho_1)e^{k_{RS}(\rho_2-\rho_1)t}} = -\frac{g_S}{g_R}\\
\iff &g_R\rho_1(\rho_0-\rho_2)-g_R\rho_2(\rho_0-\rho_1)e^{k_{RS}(\rho_2-\rho_1)t} = -g_S(\rho_0-\rho_2)+g_S(\rho_0-\rho_1)e^{k_{RS}(\rho_2-\rho_1)t}\\
\iff &(g_R\rho_1+g_S)(\rho_0-\rho_2) = (g_R\rho_2+g_S)(\rho_0-\rho_1)e^{k_{RS}(\rho_2-\rho_1)t}\\
\iff &\frac{(g_R\rho_1+g_S)(\rho_0-\rho_2)}{(g_R\rho_2+g_S)(\rho_0-\rho_1)} = e^{k_{RS}(\rho_2-\rho_1)t}\\
\iff &t_{min} := t = \frac{1}{k_{RS}(\rho_2-\rho_1)}\ln\left(\frac{(g_R\rho_1+g_S)(\rho_0-\rho_2)}{(g_R\rho_2+g_S)(\rho_0-\rho_1)}\right)
\end{aligned}
$$

We note that in the case where $N(t)$ grows monotonically (i.e., when the origin is an unstable node), the global minimum occurs at $t = 0$, so we take $t_{min} := 0$. If $N(t)$ decays monotonically (i.e., the origin is a stable node), t_{min} is not well defined. For each of Cases A, B, C, and D, we find that as a function of drug dose, t_{min} strongly correlates with t_P (S1-S4 Figs). We have that $t_P \approx 2t_{min}$ at low drug doses, and $t_P \approx 3t_{min}$ at high drug doses (S1-S4 Figs).
