# Supplementary material for: A model for the intrinsic limit of cancer therapy: Duality of treatment-induced cell death and treatment-induced stemness
Source: PLoS Comput Biol. 2022 Jul 25;18(7):e1010319. doi: 10.1371/journal.pcbi.1010319 (PMC9352192; doi:10.1371/journal.pcbi.1010319)
Supplement: Attachment — Submitted filename: Angelini_PLOS_CB_response.pdf [file pcbi.1010319.s008.pdf]

Dr. Feilim Mac Gabhann and Dr. Jason Papin
Editors-in-Chief
PLoS Computational Biology journal

April 21, 2022

Dr. Mac Gabhann and Dr. Papin,

Thank you for your consideration of our article "**A model for the intrinsic limit of cancer therapy: duality of treatment-induced cell death and treatment-induced stemness**" that we submitted last November to the *Public Library of Science* (*PLoS*) *Computational Biology* journal. In response to your decision we now resubmit a revised manuscript in which we think we have addressed all the reviewers' comments. We thank the reviewers for their thoughtful comments which have improved the quality of the manuscript.

In accordance with the reviewers' comments, the major revisions that we have made to our manuscript can be summarized as follows:

- By the suggestion of Reviewer #1, we have simplified the presentation of our results in the section "Parameter search" (Figure 4), in which we analyze how time to progression (TTP) depends on the efficacy (E_SR) and potency (P_SR) of a drug to induce resistance in cancer cells. Instead of plotting the TTP at every drug dose for different values of E_SR and P_SR, we simplify the plots in Figure 4 by plotting the following three "key" features of the TTP curve as functions of E_SR and P_SR: drug dose at which TTP is maximized, maximum TTP, and TTP at MTD. We have updated the narrative in accordance with this new figure.

- In response to several of the reviewer's comments that our model was not grounded in clinical data, we have performed a "virtual patient cohort" simulation because of the lack of clinical data that would be suited to test our model. In the simulation we assume that key parameters of our model are not fixed but vary from patient to patient. The results from these simulations are summarized in a new figure, Figure 5, showing the survival fraction of a patient cohort as a function of time. Although we do not go as far as to fit our model parameters to data, the analytical framework presented sets up a clear path forward in this direction.

Below, we give a point-by-point response to the specific comments made by the reviewers, mentioning any revisions that we have made to the manuscript.

Sincerely,

Erin Angelini
PhD Candidate, Department of Applied Mathematics
University of Washington
eang@uw.edu

Co-authors:
Dr. Yue Wang (Institut des Hautes Études Scientifiques, Bures-sur-Yvette, France)
Dr. Joseph X. Zhou (Immuno-Oncology Department, Novartis Institutes, Cambridge, MA)
Dr. Hong Qian (Department of Applied Mathematics, University of Washington)
Dr. Sui Huang (Institute for Systems Biology, Seattle, WA)

<u>Response to reviewer #1:</u>

1. *Authors note that Figures 3A & 3C are non-monotonic in lambda 1. Is this true? It's difficult to tell visually from the figures, as it appears that lambda 1 plateaus to a lower bound. In this case, it might be helpful to pick an exaggerated example.*

<u>Response:</u> We agree the non-monotonicity was hard to see, thus, in the revision we have included insets on these plots that better show the non-monotonic nature of the eigenvalue lambda_1.

2. *Can the authors please describe the reasoning behind displaying the summation "kSR + kRS" in figure 3? This represents the rate of flow between Resistant/Sensitive, added to the rate of flow between Sensitive/Resistant. Is this correct, and why is the addition an important quantity (and not, for example, the net total rate of Sensitive: gS - kRS). If I understand correctly, kRS is a constant (not a function of drug dose), as it does not appear in figure 2.*

<u>Response:</u> We plot the quantities k_SR+k_RS and g_S+g_R in Figure 3 to emphasize how the difference between these two quantities (as a function of drug dose) sets the shape of the TTP curve. As summarized in Table S1, the net growth rate of sensitive cells (g_S-k_RS) effectively governs whether or not the origin is a saddle point. This "information," however, is already summarized in the TTP plot – the origin is necessarily a saddle point wherever TTP is nonzero and finite. As such, towards the end of illustrating how the phenotype switching dynamics affect TTP, we hold that k_SR+k_RS and g_S+g_R are more informative quantities to visualize than g_S-k_RS or g_R-k_SR.

It is correct that, in this model, k_RS is a constant and does not change with drug dose. We emphasize this assumption in the revised manuscript for clarity.

3. *I think it would be helpful to place at least one of the supplementary figures (S1 through S4) inside the main text, to show example trajectories under treatment.*

<u>Response:</u> In the original manuscript, we did not include these supplementary figures in the main text, because we already included an example trajectory of the tumor size under treatment in Figure 1. We have made no changes to this figure in the revised manuscript.

4. *Figure 4 is a nice result, although I wonder if the result can be simplified. Perhaps this simulation could be simplified for plotting the dose at which max value of TTP occurs, across the range of values for PSR and ESR. If the corresponding dose is equivalent to MTD (100), then it's implied to be monotonic.*

<u>Response:</u> We have revised Figure 4 in accordance with Reviewer #1's suggestions. The new figure includes three plots (Panels A, B and C) in which the following quantities are represented by color in the (E_SR , P_SR) plane: (A) the dose at which TTP is maximized, (B) the maximum value of TTP, and (C) the value of TTP at MTD. We have also revised our presentation and analysis of the results to reflect the new figure.

5. *The point in the discussion about adaptive therapy is intriguing. While it is certainly true that the MTD dose for cases A & C result in inferior TTP, it does not necessarily follow that adaptive therapy would outperform MTD here, because adaptive approaches often administer MTD dosing, but with periodic holidays. Perhaps it would be good to note the worthwhile treatment options of both adaptive therapy and metronomic, or other dose minimization strategies, given that there appears to be an optimal dose, m, in cases A & C.*

Response: We revised the final paragraph in the Discussion section as follows (changes highlighted in red) to refer to other dose-minimization strategies, as opposed to just adaptive therapy.

> *"Adaptive therapy, which stops treatment upon regression and re-starting if there is regrowth, or metronomic therapy, which applies a low dose at regular intervals, may be worthwhile treatment schemes in cases A and C because there is an optimal drug dose below MTD in these cases. The current rationale behind dose-minimization treatment strategies is to avoid fixation of mutant resistant clones due to competitive release and selection. However, if resistance is inducible and reversible, the intended ``containment'' of the tumor (as opposed to the harder-to-achieve ``eradication'') is even more possible than it is under the pretext of competitive release of mutant resistant clones. Thus, if we consider the new biological rationale of non-genetic, reversible dynamics of treatment-induced resistance, a strategy such as adaptive or metronomic therapy may be even more effective than currently understood. …"*

6. *Please place a box around the legend in Figures S1-4 (right side of panel A), because it makes it appear that the legend marker is a data point which doesn't fall on the black line.*

Response: We have updated the supplementary figures to include boxes around the legends.

Response to reviewer #2:

1. *How the dynamics of the model compares to similarly simple models, which involve just competition between resistant and sensitive cells, without invoking induction of stemness and resistance. It will be interesting and important to show that this model results in dynamics different from the almost equally simple nonlinear ODE model of lung cancer treatment by Kozlowska et al. (PLoS CB 2020), which involves only selection of pre-existing resistant cells. Will such not lead to trajectories similar as in the "saddle-point" set-up?*

Response: The reviewer raises an interesting point. At the surface level, a competitive Lotka-Volterra model (such as that used by Kozlowka *et al.* in their 2020 PLOS CB paper) that only considers selection of pre-existing resistant clones results in similar dynamics as our linear model with phenotype switching: when the net growth rate of one population is larger than that of the other, the origin becomes a saddle point, and we get tumor dynamics of depletion and progression. We have added the following sentence to the first paragraph in the discussion section to acknowledge this similarity: "*Nonlinear models of competition, such as that presented by Kozlowska* et al.*, lead to similar saddle-point dynamics of tumor depletion and progression as our model*."

The selection mechanism, however, operates at a longer time-scale than the mechanism of induced resistance, as the population-level phenotype switch to drug-resistance requires multiple generations. By contrast, one motivation of our model was to explain the oft observed appearance of population wide resistance within one cell generation time. In reality, both mechanisms contribute to drug resistance. A future model will combine both induction and selection mechanisms at appropriate weight. The addition of non-linear competition terms to our model, whether in the form of direct competition or indirect competition via a shared resource, would likely have a significant effect on the long-term behavior of solutions. By contrast, our model analysis is focused in large part on a transient aspect of solutions: time to progression.

Here, we postulate and show that the unique behavior of our model (the non-monotonic TTP curves) comes from the inclusion of the phenotype switching term. Our assumption is that the relation between the sigmoid dose-response curves d_S(*m*) (sensitive cell death), d_R(*m*) (resistant cell death), and k_SR(*m*) (induced resistance) is what causes TTP to first increase then decrease with drug dose. Specifically, we find that the TTP is a non-monotonic function of drug dose *m* when a drug has a lower potency (i.e., a higher dose is needed to reach the maximum effect) to induce resistance than it does to kill sensitive or resistant cells. This scenario creates a dose regime at low doses in which cell growth dominates over the induction of cell state resistance, and a regime at intermediate doses in which the induction of resistance dominates over cell growth (Fig 2, left column; Fig 3 A & C). These two regimes are regimes in which TTP is increasing and decreasing with drug dose, respectively (Figure 3 A&C). Figure 4 highlights that these dynamics are robust to our choice of efficacy to induce resistance (k_SR at MTD) – when potency to induce resistance is high, the non-monotonic behavior disappears (Fig 4A). Thus, inclusion of phenotype switching and the assumption of the sigmoid shape of the dose-response curves are integral to the results presented in this model, regardless of if our ODE model is linear or not.

2. *Enough is known by now about progression dynamics of many types of cancer under treatment, to endow the model with parameters more thoroughly grounded in data. Kozlowska et al. paper on lung cancer has such estimates, and an earlier paper in Cancer Research with the same first author features estimates for ovarian cancers. The same is the case regarding another Cancer Research paper from Bozic lab concerning colon cancer. Which of the 4 regimes will prevail under data from these papers?*

<u>Response:</u> The reviewer asks an interesting and important question about the practicality of our model analysis. This comment is partially addressed by our addition of the virtual patient cohort (VPC) simulations and the resulting Kaplan-Meier curves to the manuscript. However, we think that it is beyond the scope of this paper to fit the model to real-world data as the reviewer envisions, albeit it will be of great scientific value. Such an exercise would require extensive data curation to exclude confounding factors because of the heterogeneity of studies. Moreover, paucity of dose escalation studies will be a major challenge in validating our results in a retrospective study of clinical trial data. One could however envision an animal model. In any case, the VPC analysis that makes a minimal set of plausible, elementary assumptions on patient treatment response demonstrates a step toward grounding our model predictions in experimental/clinical data. In the revised manuscript we also refer to the suggested papers in the discussion section for estimates of some of our model parameters, and we note that using these empirical estimates is one possible way to incorporate data into our model.

We also repeated our model analysis with a more realistic initial tumor size (10^9) than was previously used (~10^2) with the same initial fraction of resistant cells (0.1). Our new figures show the

results of this analysis. Interestingly, little actually changed in the resulting time to progression curves, indicating that TTP is more sensitive to the initial tumor composition rather than the size.

3. *In the older literature, gradual evolution of resistance was considered, such as resistance to methotrexate induced by DHFR gene amplification. How will performance of the current model change under gradual evolution of resistance? A thorough study would require far-reaching generalization of the model, but it is possible that relevant mathematical results exist in the literature. It seems fair to request at least a discussion of these issues.*

Response: We agree that the historical, rather idiosyncratic but fascinating case of DHFR gene amplification as a mechanism of resistance may represent a more profound similarity worthy of a detailed comparison. Superficially, it is different: (1) In the DHFR case, we have a genetic process that depends on genomic alterations, which in general are considered stochastic in nature (although there is increasing evidence that they are not); (2) DHFR amplification is a multi-step process in which the resistance gradually increases with the increase of copy numbers and duration of treatment; (3) The individual steps in the DHFR case are coupled to cell division and take place at a longer time scale.

Here we study a non-genetic process due to phenotypic plasticity and consider one single step. However, at a more formal level, there are interesting similarities between these two resistance mechanisms beyond the specific molecular mechanism: the initiation of amplification (which underlies resistance) can be drug-induced (chromosome breakage) and is also more prone in cancer cells – similar to the induction of the stem-like state that confers resistance in our work, a behavior for which cancer cells exhibit an increased proclivity. The increased phenotypic plasticity with increasing malignancy is also well known, perhaps due to the increased number of (parallel) state transition paths associated with increased immaturity, affording an increasing combinatorial diversity for the entry into the drug-resistant state – this is another parallel to the DHFR case which has been modeled using a branching process model.

We now discuss this interesting historical aspect and potential generalization of the problem of multi-step increase in resistance – at the cost of extending the discussion by a paragraph; herein we also cite the relevant work that may be of interest to readers who take a broader, more generalized view (Kimmel et al, 1992).

4. *The paper is generally very well-written, although the term "baked-in" seems a bit idiosyncratic compared to the more common "wired-in".*

Response: We have updated the wording used in the manuscript in accordance with this comment.

Response to reviewer #3:

1. *I always appreciate working with a minimal semi-mechanistic model. What I wonder here is: is this model too simple to provide meaningful predictions? I would be less concerned about the simplicity of the model if there was some calibration/validation against an experimental dataset. But, in the absence of this, I'm left wondering: 1) is the assumption of exponential growth sufficient, and 2) are the functional*

forms in eqn. (4) reasonable choices to model the impact drug dose has on the rates of cell-kill and transition-to-resistance?

Response: In response to question 1), we agree that in general, having something along the lines of logistic growth (i.e., a nonlinear model) would better model the long-term dynamics of the tumor – with our model, solutions tend to infinity under the saddle point conditions, which is clearly unrealistic. However, our model analysis of time to progression hinges on the *transient* dynamics of such solutions and we focus on the initial "take-off" of tumor cell populations inspired by cell culture and animal experiments in the laboratory (manuscript in preparation, models based on our previous work, see e.g. PMID: 29191914 and PMID: 24045430). Hence, our assumption of exponential growth (i.e., a linear model) is "sufficient" because it is roughly how logistic growth solutions behave in the short-term. We treat the problem of assuming a linear versus a non-linear model more thoroughly in our response to Reviewer #2's first comment.

In response to question 2), we hold that our assumption that the dose-response curves (d_S, d_R, k_SR) have a sigmoid shape, which is a standard pharmacodynamic assumption; this captures both "zero-order ultra-sensitivity" (due to Goldbeter and Koshland), which is ubiquitous in non-elementary multi-step biological processes, and saturation. There are several functional forms (e.g., Hill-type function, tanh) that also achieve sigmoid shapes, and we could have just as well chosen these alternative forms, which share the key features of half-maximum inflection point and a maximum value. Our analysis would not have been significantly altered qualitatively by using a different functional form for the sigmoid curves.

2. *The TTP analysis is very nicely presented. I guess I'm just stuck wondering: what has been gained from the analysis? I absolutely think the message that designing a dosing strategy cannot be optimally done without understanding the propensity of the drug to induce resistance is an important one. But, if one accepts the evidence of drug-induced resistance, is it not self-evident that optimal dosing strategies would be influenced by this drug-induced phenotypic transition? If the model were grounded in data, that would be different, as then directly actionable suggestions could be made. But without that, the result really is: induced resistance can impact TTP. No doubt I agree, but I agreed with this point just from reading the intro to the paper!*

Response: We thank the reviewer for sharing this particular viewpoint, and fully agree, notably that *"the message that designing a dosing strategy cannot be optimally done without understanding the propensity of the drug to induce resistance is an important one."* This point deeply resonates with our intention. However, the evidence for direct induction (not selection) of a phenotypic change of the cell (a true conversion of the very same cell, not death and replacement) has only been obtained in recent years, made possible by single-cell resolution cell tracing. The goal of this present work is to provide a first, generic elementary quantitative analysis, in the sense of a systematic evaluation of hypothetical scenarios that considers this new biological insight. It is clear that one day we must consider this biological phenomenon in "designing the dosing strategy," as the reviewer states. However, this will require much more data from a distinct type of preclinical studies that is currently almost never performed: detailed dose response measurements that not only measures cell death as a function of drug dose but also the qualitative changes in the *surviving* cells.

Thus, at this point, the scope of this paper is a first presentation of an analytical and qualitative analysis of a simple model for non-genetic, drug-induced resistance in cancer, free of unnecessary details so as to promote a better conceptual understanding of the impact of this phenomenon on tumor growth dynamics. One of our takeaways is that induced resistance not only affects TTP, but can be a limiting factor on treatment success.

Thus, we acknowledge that we cannot make any specific clinical prediction based on our current model, as our model parameters are not informed by any empirical estimates. Hence our emphasis on the parameter space - which resulted in the”two levels” of parameters (Fig 1). However, to facilitate future grounding of our (or a similar) model in clinical data, we have added a section in which we simulate virtual patient cohorts by drawing a set of our parameters from a probability distribution. From these simulations, we generate Kaplan-Meier survival curves, which are more clinically relevant measures of treatment success than computing the TTP for an individual patient. While fitting our model to clinical data is a possible future direction of research, such a study is beyond the scope of this paper.

3. *Many of the qualitative conclusions made seem fairly intuitive, given the assumptions built into the model. While more quantitative aspects of the conclusions are certainly not intuitive, those are also a lot more dependent on functional form and the somewhat arbitrary values parameters were set to. In particular, the following did seem intuitive without an analysis of the model: 1) In the case of high efficacy of induction (Cases A, B), there is diminishing TTP gains as done increases. 2) Saturation level of TTP decreases as efficacy of resistance induction increases. The one result I found less intuitive is that non-monotonicity of TTP is only observed when the potency to induce drug resistance is low. Though, I do think it's more accurate to say "low relative to the potency of tumor killing".*

Response: Similar to our statement in the previous point, we are pleased to see that the reviewer finds our result intuitive or somewhat expected. However, it may not be intuitive to many readers in the wider cancer research community, given deeply ingrained preconceptions to achieve maximal cytotoxic dose (“The more the better”). Clearly, the principle that intensive treatment may “backfire” - is not at all appreciated, not even in the context of competitive release in the case of selection dynamics. Our goal is to formalize the alternative non-genetic, non-selection mechanism, and show that the non-monotonic effect is relatively robust, i.e., it is not a singular, idiosyncratic behavior limited to a narrow space conditions but can occur in a relatively broad parameter regime. The existence of the latter will be qualitatively intuitive to the reader who have been exposed to modeling drug response dynamics.

As stated by now a few times (in response to other concerns above), the goal of this manuscript is thus not to report a “surprising result,” but to offer a first formal analysis of the impact of recently accumulating experiment observations of treatment-induced conversion to a more resistant cell phenotype. In doing so, we aim to complement existing theoretical work on other mechanisms of drug resistance that underlie the need to think beyond “the more is better” (in view of anti-cancer drug dose, limited solely by toxicity), which for instance has motivated adaptive therapy.

In regards to the wording of “low potency” of inducing resistance (P_SR) being relative to the potency of tumor killing (P_S and P_R), we have updated the manuscript in response to this helpful suggestion.

4. *Lines 78-79: the authors state that “However, the intrinsic limit that induced resistance places on therapy has not been systematically evaluated.” While not as systematic analysis as done herein, this was*

studied in a model of induced resistance in "Mathematical Approach to Differentiate Spontaneous and *Induced Evolution to Drug Resistance During Cancer Treatment" by Greene and colleagues.*

Response: We appreciate this suggestion and have revised the sentence in question to say "*...has only recently been systematically evaluated*" with a reference to the paper by Greene *et al*.

5. *Figure 1: why isn't the drug dose m shown influencing the death of resistant cells?*

Response: We do not include plots of all three parameters (d_S, d_R, k_{SR}) that change with drug dose in Figure 1 because this figure is meant to illustrate the overall schematic of our model. We wanted to keep the figure simple and uncluttered, hence only showing an example of a sigmoid dose-response curve in Figure 1. Instead, we plot d_S, d_R, and k_{SR} as functions of drug dose *m* in Figure 2.

6. *At first I was having trouble interpreting "high potency" and "low potency". Further (and more careful) reading made clear that high potency means that a low concentration of drug is needed to cause the halfway response between baseline induction and maximal induction. While it makes sense to call this "high potency", I found it confusing and thought the language could be clarified earlier in the paper.*

Response: We realize that this can be confusing and now provide an explanation of potency (as formally defined in pharmacology) and have updated the wording in the manuscript to clarify the inverse relationship between EC50 and "potency." Specifically, we revised the third paragraph of the "Pharmacodynamic model…" section, with changes highlighted in red:

> *"In the case of* $d_S(m)$*, each of the four parameters* δ_S, E_S, r_S *and* P_S *determines the shape of the logistic curve and describes a behavior affected by the drug. For this reason, we refer to these parameters as the* pharmacodynamic parameters *associated with a given rate constant for cell response. In particular, the parameters* E, P, *and* r *respectively determine the maximum response* (efficacy)*, EC50 (which is inversely related to* potency)*, and saturation rates for each of the above responses of the drug. For example, a drug with high efficacy and high potency to kill sensitive cells is characterized by a high value for* E_S *and a low value for* P_S*."*

7. *Line 226: "We first consider a drug with a high efficacy and low potency (i.e., high EC50) for inducing resistance to cell killing". At first the language threw me off, because there are also terms in the model that describe the efficacy and potency of cell killing. It would be clearer to me if this just said "We first consider a drug with a high efficacy and low potency (i.e., high EC50) for inducing resistance."*

Response: Similarly, we have revised the wording in the manuscript to improve clarity as suggested in this comment.

8. *I was wondering if the authors could explain, in Figure 3 (bottom row), why all four plots seem to first hit their maximum value around the same dose of m = 40. I would have thought that the potency parameter* P_{SR} *would have influenced this time, but maybe it's more a function of the fixed parameters in the model?*

Response: The parameter P_SR does indeed influence where TTP attains its maximum. Looking at the plots of for (k_SR+k_RS) (Figure 3, top), this “optimal”drug dose is also the dose at which the value of (k_SR+k_RS) starts to rise with increasing dose *m*. By the nature of the sigmoidal shape of k+SR as a function of m, we would expect this point to be just before the EC50, P_SR. The exact point is also influenced by the parameter r_SR, which controls the steepness of the sigmoid curve (see Table S2).

9. *When I downloaded the supplemental figures, they were of low image quality, even when viewing them in a small window.*

Response: We have updated the supplementary figures to 600 dpi resolution.
